# Difficulty in diagnosing peritonitis caused by multidrug‐resistant tuberculosis

**DOI:** 10.1002/ccr3.7759

**Published:** 2023-08-29

**Authors:** Sayaka Aoyama, Shun Yamashita, Kosuke Ishizuka, Shinichi Katsukura, Hiroki Matsuura, Mikiro Kato

**Affiliations:** ^1^ Department of Internal Medicine, Mito Kyodo General Hospital University of Tsukuba Ibaraki Japan; ^2^ Department of General Medicine Saga University Hospital Saga Japan; ^3^ Department of General Medicine Chiba University Hospital Chiba Japan; ^4^ Department of Diagnostic and Generalist Medicine Dokkyo Medical University Hospital Tochigi Japan; ^5^ Department of General Internal Medicine Okayama City Hospital Okayama Japan; ^6^ Department of Infectious Diseases University of Tsukuba Ibaraki Japan

**Keywords:** abdominal contrast‐enhanced computed tomography, multidrug‐resistant tuberculosis, peritoneal biopsy, peritonitis

## Abstract

**Key Clinical Message:**

The low sensitivity of ascites culture for acid‐fast bacilli necessitates a peritoneal biopsy when tuberculous peritonitis is suspected. Findings in the peritoneum on computed tomography may prompt suspicion of tuberculous peritonitis.

**Abstract:**

A 47‐year‐old Nigerian man presented with fever, abdominal distention, and weight loss. Abdominal computed tomography revealed massive ascites and peritoneal thickening. Despite failing to culture acid‐fast bacilli from ascites, histological examination and culture of peritoneum revealed multidrug‐resistant tuberculosis peritonitis. Peritoneal biopsy is mandatory when tuberculosis peritonitis is suspected.

## Graphical abstract

Findings of abdominal contrast‐enhanced computed tomography in patients with tuberculous peritonitis.

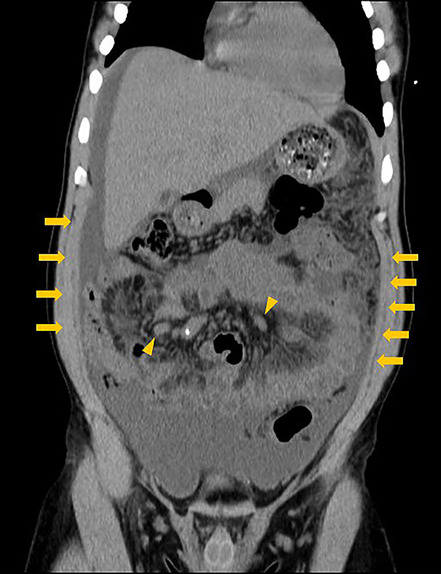

A 47‐year‐old Nigerian man who had been living in Japan for 20 years and had visited to Nigeria 3 years previously presented with lost 15 kg of body weight accompanied by abdominal distention without tenderness over 2 months. He had had a fever of over 38°C and watery diarrhea for 1 month. On admission, abdominal contrast‐enhanced computed tomography showed massive ascites, homogeneously thickened peritoneum, and enlarged mesenteric lymph nodes (Figure 1). Examination of a sample of ascites revealed abundant white blood cells (1483/μL; lymphocytes: 85.0%), a high adenosine deaminase concentration (128 IU/L), culture‐negative acid‐fast bacilli (AFB), and a negative polymerase chain reaction for *Mycobacterium tuberculosis*. Laparoscopy showed white miliary nodules and fibrous adhesions (Figure 2A,B). Histologic examination of a peritoneal biopsy showed caseating granulomas and Langhans giant cells (Figure 2C,D). Culture of a peritoneal sample revealed *M. tuberculosis* that was resistant to rifampicin, isoniazid, and streptomycin. Thus, he was diagnosed with tuberculous peritonitis (TBP) and transferred to a hospital that specializes in the treatment of tuberculosis.

**FIGURE 1:**
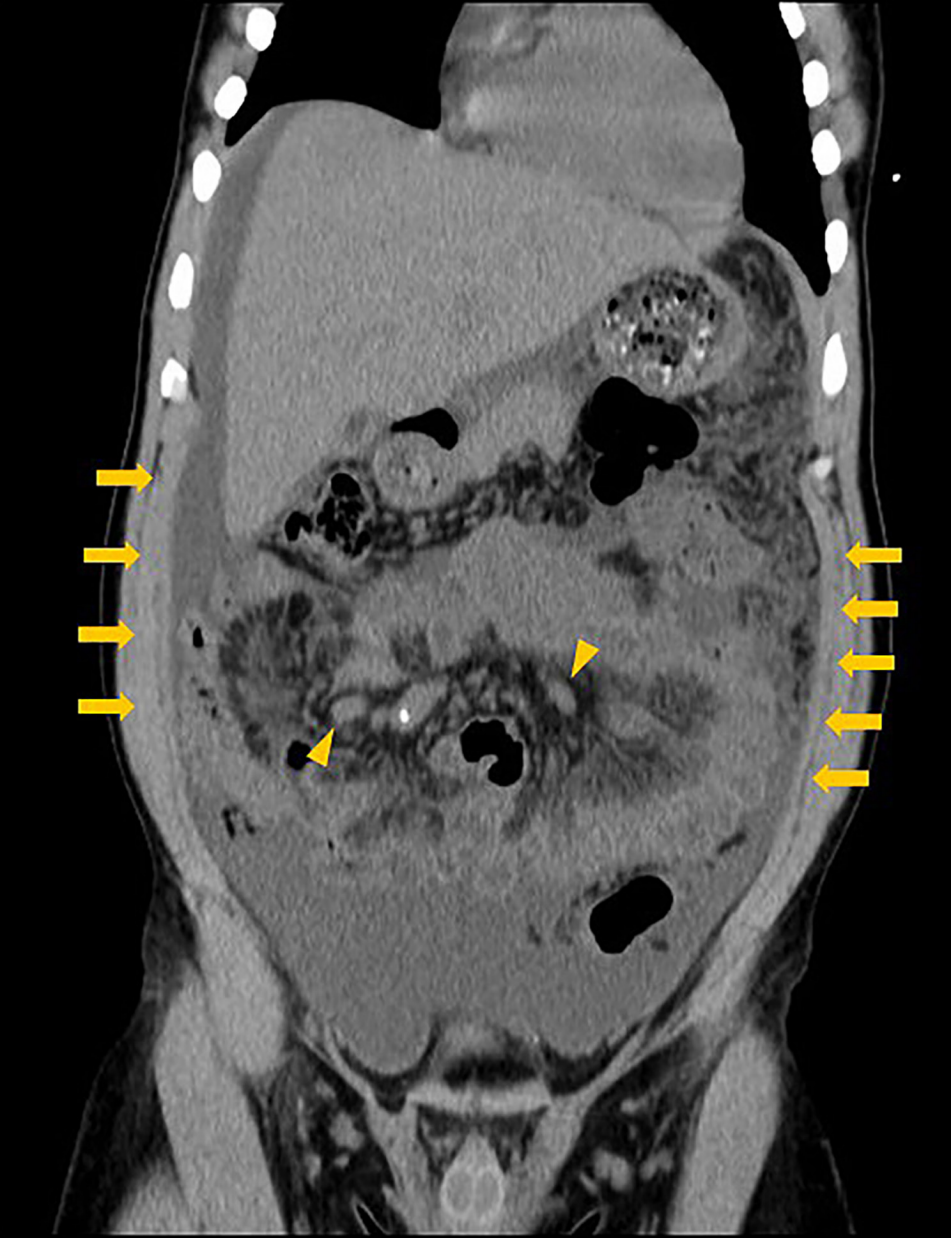
Findings of abdominal contrast‐enhanced computed tomography on admission. There is massive ascites, diffuse, smooth, homogeneous thickening of the peritoneum (arrows) and enlarged mesenteric lymph nodes (arrowheads).

**FIGURE 2:**
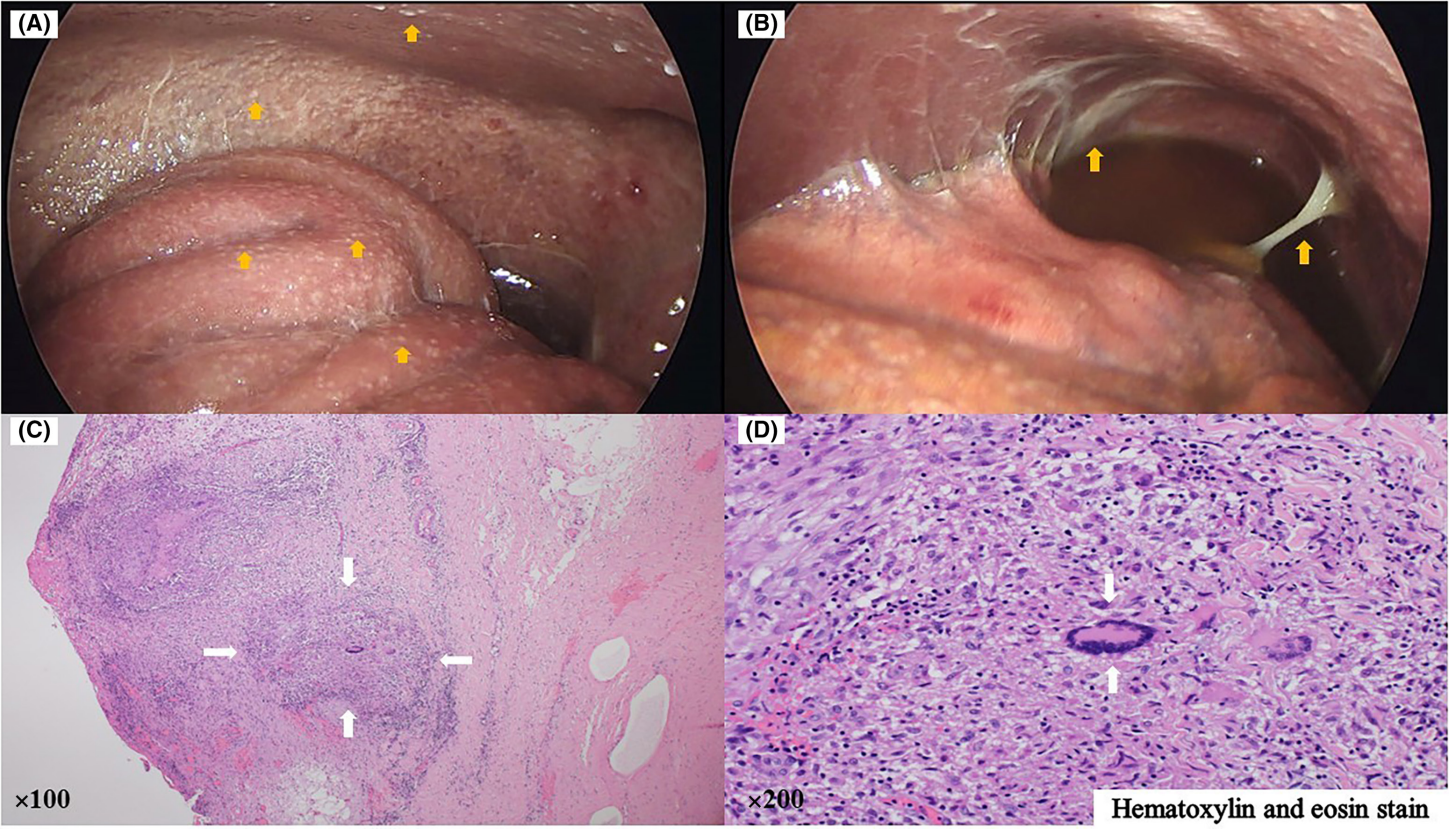
Findings on laparoscopy and histologic examination of a peritoneal biopsy. (A) White miliary nodules. (B) Fibrous adhesions. (C) Caseating granulomas (hematoxylin and eosin staining, ×100). (D) Langhans giant cell (Hematoxylin and eosin staining, ×200). Laparoscopy revealed numerous white miliary nodules (arrows, A) and fibrous adhesions (arrows, B), which are suggestive of peritoneal tuberculosis and peritonitis, respectively. Photomicrograph of peritoneal sample showing caseating granulomas (arrows, C) and Langhans giant cells (arrows, D), which are also suggestive of tuberculous peritonitis.

Detection of caseating granulomas in a peritoneal biopsy is required to diagnose and treat TBP because AFB are only cultured from ascites in 35% of such patients.^1^ However, it is difficult to remember to include TBP in the differential diagnosis when AFB are not cultured from ascites and/or *M. tuberculosis* is not detected by polymerase chain reaction. Diffuse, smooth, and homogeneous peritoneal thickening on computed tomography together with a high adenosine deaminase concentration in ascites should prompt suspicion of TBP.^2^ The present patient had no known previous contact with individuals with tuberculosis. However, tuberculosis is highly endemic in Nigeria, where its incidence of 4.6% is lower than only those in India, China, Indonesia, Philippines, and Pakistan. There are reportedly many patients with untreated or multidrug‐resistant tuberculosis in Nigeria.^3^ It is important to recognize that individuals from areas in which tuberculosis is endemic, such as Nigeria, may have multidrug‐resistant tuberculosis. Peritoneal biopsy and culture for mycobacterium should be performed when TBP, especially that caused by drug‐resistant tuberculosis, is suspected.

## AUTHOR CONTRIBUTIONS

**Sayaka Aoyama:** Conceptualization; data curation; resources; writing – original draft. **Shun Yamashita:** Conceptualization; supervision; writing – original draft; writing – review and editing. **Kosuke Ishizuka:** Conceptualization; supervision; writing – review and editing. **Shinichi Katsukura:** Conceptualization; supervision; writing – review and editing. **Hiroki Matsuura:** Conceptualization; supervision; writing – review and editing. **Mikiro Kato:** Conceptualization; data curation; resources; supervision.

## FUNDING INFORMATION

No specific grant was received for this work from any funding agency.

## CONFLICT OF INTEREST STATEMENT

The authors state that they have no conflict of interest.

## ETHICS STATEMENT

This manuscript conforms to the provisions of the Declaration of Helsinki in 1995 (as revised in Brazil 2013).

## CONSENT

Written informed consent was obtained from the patient to publish this report in accordance with the journal's patient consent policy.

## Data Availability

The data that support the findings of this study are available from the corresponding author upon reasonable request.
